# Update on clinimetric assessments in juvenile dermatomyositis: conceptual foundations, current tools, and future directions (Narrative review describing the development, validation, and application of clinimetric outcome measures in juvenile dermatomyositis)

**DOI:** 10.1186/s12969-026-01233-4

**Published:** 2026-06-08

**Authors:** Charalampia Papadopoulou, Brian M. Feldman

**Affiliations:** 1 Department Paediatric Rheumatology, Great Ormond Street Hospital for Children, NHS Foundation Trust, London, WC1N 3JH UK; 2 Infection, Immunity and Inflammation Research and Teaching Department, UCL Great Ormond Street Institute of Child Health, London, UK; 3 Division of Rheumatology, Department of Pediatrics, The Hospital for Sick Children, University of Toronto, Toronto, ON Canada; 4 Child Health Evaluative Sciences, SickKids Research Institute, Toronto, ON Canada; 5 Institute of Medical Science, University of Toronto, Toronto, ON Canada; 6 Institute of Health Policy Management & Evaluation, Dalla Lana School of Public Health, University of Toronto, Toronto, ON Canada

**Keywords:** Juvenile dermatomyositis, Clinimetrics, Muscle strength, Skin disease, Patient-reported outcome measures, Composite disease activity indices, Treat-to-target

## Abstract

**Background:**

Juvenile dermatomyositis (JDM) is a complex multi-system disease that significantly impacts the health and development of affected children. Accurate and comprehensive measurement of disease activity and outcomes is essential for effective clinical management and research. Traditional unidimensional measures are insufficient for capturing the multifaceted nature of JDM, necessitating the development of specialized clinimetric tools that integrate multiple health domains and perspectives.

**Main body:**

Over the past decades, significant advances have been made in developing and validating clinimetric assessments for JDM. Core domains such as muscle strength, skin involvement, global disease activity, and patient-reported outcomes are now routinely evaluated using established instruments like the Childhood Myositis Assessment Scale, Manual Muscle Testing, and various skin assessment tools. Composite indices, including the Juvenile Dermatomyositis Activity Index and the ACR/EULAR Total Improvement Score, enhance sensitivity to clinical changes by integrating multidimensional data. Imaging modalities such as magnetic resonance imaging and muscle ultrasound provide objective insights into muscle inflammation and damage, while histopathology scoring offers valuable research and prognostic information. Despite these advances, challenges remain in standardizing assessments, particularly for skin disease and patient-reported symptoms like fatigue and social participation. Emerging digital health technologies and biomarker integration hold promise for improving longitudinal monitoring and precision in disease evaluation. Additionally, the treat-to-target approach in JDM underscores the need for frequent, accurate measurements to guide therapy adjustments. However, practical barriers including resource limitations and tool complexity hinder widespread clinical adoption.

**Conclusion:**

Considerable progress has been achieved in the development of clinimetric tools for JDM, enabling more nuanced and patient-centred disease assessment. Future efforts should prioritize the creation of disease-specific patient-reported outcome measures, streamlined and feasible assessment protocols, and the integration of biological markers and digital innovations. These advances will support precision medicine approaches and improve outcomes for children living with JDM.

**Clinical trial number:**

Not applicable.

## Background: why do we measure in JDM? What are we measuring?

JDM is a multi-system disease that significantly affects well-being and development. Due to clinicians’ and researchers’ tendency towards confirmation bias [1–4], it is crucial to use relatively objective measures of patients’ health for accurate management.

Historical ways of measuring properties (e.g., simple/unidimensional physical properties like mass or distance) don’t work for complex phenomenon (e.g., multi-dimensional concepts like “health”, or “well-being”). The development of conjoint measurement axioms [5] provided theory supporting multi-item questionnaires for psychological properties (supporting the development of “psychometrics”, e.g. IQ tests). Alvan Feinstein [6], recognizing that the psychometric approach doesn’t address the complexities of many health conditions (in which some aspects might improve while others worsen), coined the term “clinimetrics” as a distinction.

It is this complexity that must be considered when measuring health in JDM.

## Principles of measurement in JDM clinimetrics

Good instruments have several properties in common. They must be valid (measure what they purport to measure), measure health aspects important to patients, cover all important health aspects, be useful (lead to better decisions), reliable (stable between observations – and between observers – for stable patients), responsive (change when the health states change), and feasible (time and resource affordable in clinical/research settings).

## Historical development of clinimetric assessments in juvenile dermatomyositis

Historically, assessment of juvenile dermatomyositis (JDM) was limited by the disease’s rarity and few treatment options, with clinicians relying on ad hoc evaluation of muscle weakness, skin findings, and laboratory results such as serum CK. These non-standardized approaches often failed to reflect the complex and variable manifestations of JDM.

With advances in paediatric rheumatology, it became clear that standardized, validated outcome measures were needed for consistent disease monitoring, research, and clinical trials. International collaborations led to two major initiatives: IMACS and PRINTO, which established core outcome domains for JDM, including muscle strength, physical function, physician and parent/patient global assessments, and extramuscular activity [7–10]. PRINTO notably excluded muscle enzymes because of their inconsistent correlation with disease activity [9, 10].

Adoption of these core sets allowed for uniform assessment and reporting. However, early response criteria, based on binary thresholds, did not capture the nuanced, asynchronous improvements in disease seen in JDM. For example, muscle strength could improve while skin symptoms or fatigue persisted [9, 10].

Over time, it became clear that clinician-reported measures alone were insufficient, prompting the inclusion of patient-reported outcomes measures (PROMs), such as the CHAQ and quality-of-life (QoL) surveys [9, 11, 12], though these lacked disease specificity and were primarily used in research [13]. This led to the development of integrated tools that combine clinician and patient perspectives, including the Juvenile DermatoMyositis Activity Index (JDMAI) [14], reflecting a shift toward more comprehensive, patient-centred assessment in JDM [10].

## Current tools and their measurement properties

### Muscle assessments

Muscle involvement is central to the assessment of JDM. The two main tools are the Childhood Myositis Assessment Scale (CMAS) and Manual Muscle Testing of 8 muscle groups (MMT8), each capturing different aspects of muscle strength and function [9, 15, 16].

CMAS is a 14-item scale evaluating muscle strength and endurance through functional tasks such as timed arm raises and sit-ups. It is reliable, valid, and sensitive to clinical changes, with scores ranging from 0 to 52 (higher scores indicate better function) [9, 11, 15–18]. CMAS is particularly valuable in paediatric settings because it measures endurance and functional capacity, but it has limitations: it may not detect mild or residual weakness (ceiling effects), focuses mainly on proximal and axial muscle groups, and performance can be influenced by motivation, fatigue, or age. Age-specific reference values are recommended due to differences in normative performance [19].

MMT8 assesses strength in 8 proximal, distal and axial muscle groups, is practical and quick to use, and requires minimal equipment [15, 20]. MMT8 scores are reported on a 0–150 scale, based on bilateral testing of seven muscle groups and the neck flexors, or on a 0–80 scale when unilateral muscle strength testing is used [20]. MMT8 aligns well with CMAS and clinician assessments but is less sensitive at higher strength levels and is prone to variability between observers, even with training [20]. Shortened versions (MMT4, MMT6) may be more feasible in some settings [21]. CMAS and MMT8 are often combined in practice.

A hybrid method (hMC) combines all MMT8 items with 3 CMAS items (head lift, abdominal strength, floor rise) for a 0–100 score, demonstrating excellent reliability and responsiveness [16].

### Skin assessments

Cutaneous involvement is a major source of morbidity in JDM, often following a disease course separate from muscle symptoms. Persistent rash, ulceration, or calcinosis can significantly impact quality of life, highlighting the importance of accurate skin assessment [22–24].

The skin component of the Disease Activity Score (DAS) provides a straightforward method for assessing rash extent and severity, including features such as Gottron papules and periungual changes; however, because it captures only a limited range of cutaneous features, it may not fully reflect the broader spectrum of JDM skin activity. DAS skin scores range from 0 to 20, and the tool is noted for its simplicity and reliability, with strong correlations with visual analogue scales of skin [9, 22].

The Cutaneous Assessment Tool (CAT), designed for inflammatory myopathies, evaluates both disease activity and permanent damage, capturing a broad spectrum of skin manifestations and distinguishing reversible from irreversible changes. Although the original CAT is relatively complex, a simplified binary method [25] based on the presence or absence of lesions has been developed; this abbreviated version remains reliable, is responsive to change, and reduces completion time, improving feasibility for routine clinical use [22, 23, 25–27].

The Cutaneous Dermatomyositis Disease Area and Severity Index (CDASI), developed originally for adult patients, quantitatively measures skin activity and damage in children as well. CDASI correlates well with CAT and physician global skin assessments, is responsive to change, and is associated with QoL measures; roughly 40% improvement in CDASI activity can reflect meaningful QoL gains [22, 23, 26–29]. However, some descriptors require further adaptation for paediatric use.

All these skin tools have similar measurement properties, correlate highly over time, but face challenges related to subjectivity, skin phototype, and limited assessment of symptoms such as pruritus or pain [30].

The key muscle and skin assessment tools used in JDM, including their measurement properties and limitations, are summarised in Table 1.

**Table 1:** Key muscle and skin assessment tools in juvenile dermatomyositis

Tool	Domain	Score range (direction)	Main construct measured	Measurement properties	Key limitations
CMAS	Muscle	0–52 (higher = better)	Functional muscle strength and endurance	Strong reliability, validity, responsiveness	Ceiling effects; influenced by motivation/fatigue; age norms needed
MMT8	Muscle	0–150 (bilateral testing of seven muscle groups and the neck flexors) or 0–80 (unilateral muscle strength testing) (higher = better)	Strength of 8 muscle groups	Good inter-/intra-rater reliability with training; core response tool	Reduced sensitivity near normal strength; observer variability
hMC	Muscle	0–100 (higher = better)	Combined proximal strength and functional performance	Excellent reliability and responsiveness	Limited uptake; requires standardisation
DAS (skin)	Skin	0–20 (higher = worse)	Rash extent and severity	Feasible; correlates with skin VAS	Coarse grading; subtle change not well captured
CAT	Skin	Separate activity/damage items	Active skin disease and permanent damage	Good reliability and construct validity	Length and complexity limit routine use; abbreviated version remains reliable, is responsive to change, and reduces completion time
CDASI	Skin	Activity and damage sub-scores (higher = worse)	Quantitative skin activity and damage	Responsive; correlates with skin PGA and QoL	Adult-derived descriptors; subjectivity; limited symptom capture

Abbreviations: CMAS, Childhood Myositis Assessment Scale; MMT8, Manual Muscle Testing of 8 muscle groups; hMC, hybrid muscle score; DAS, Disease Activity Score; CAT, Cutaneous Assessment Tool; CDASI, Cutaneous Dermatomyositis Disease Area and Severity Index; PGA, Physician Global Assessment; QoL, quality of life

### Global assessments and laboratory measures

Physician and parent/patient global assessments are core measures in JDM, integrating overall disease activity from both clinical and patient perspectives [9, 10, 15]. Physician Global Assessment (PGA) reflects a synthesis of muscle, skin, and systemic features, but inter-observer variability exists. Parent/patient global assessments capture symptoms like pain and fatigue, often differing from clinician assessments and highlighting the need for both perspectives [12, 31]. Parent/patient global assessments provide essential insight into symptoms such as pain, fatigue, and psychosocial impact that may not be fully appreciated during the clinical examination. Discrepancies between physician and parent perspectives are common and underscore the importance of integrating both perspectives into disease assessment [12, 31].

Muscle enzymes such as CK and aldolase are commonly used but often poorly correlate with disease activity; normal enzyme values do not rule out active disease, and enzyme elevations do not always indicate ongoing myositis-related inflammation, as they may reflect non-inflammatory or non-muscle factors such as strenuous exercise, genetically or ethnicity-associated higher baseline CK levels, or elevations in transaminases or LDH arising from liver or lung involvement [32, 33]. More than 20% of children may have normal CK at diagnosis, and enzyme values can normalise with corticosteroid treatment even in ongoing active disease [33]. As a result, enzymes are best considered supportive measures interpreted within the broader clinical context. Newer biomarkers, including galectin-9 and CXCL10, may outperform CK in detecting active disease and are promising for future multidimensional assessment [33, 34].In addition, type I interferon pathway measures are increasingly being explored for disease monitoring, with recent data suggesting that IFN-β may reflect muscle disease activity across JDM subgroups, whereas IFN-α may have particular relevance in anti-MDA5-positive JDM [35]. Anti-MDA5 antibody titres may also track disease severity, particularly ILD and cutaneous activity, although their role in routine monitoring remains to be standardised [36]. Circulating endothelial cells have similarly been proposed as biomarkers of endothelial injury and vasculopathy in JDM, correlating with active disease and extramuscular activity in some cohorts [37, 38].

### Imaging assessments

Imaging, especially MRI, is increasingly important for diagnosing and monitoring JDM, detecting both active inflammation and chronic changes. STIR or fat-suppressed T2 sequences sensitively detect muscle oedema indicative of active inflammation [39–42]. MRI can also identify chronic changes such as fatty infiltration or atrophy, aiding differentiation between active disease and damage [42]; it can additionally detect fascial and subcutaneous oedema [43], including clinically occult involvement, with abnormal subcutaneous fat signal at presentation associated with later calcinosis [43] and a more chronic disease course [44]. However, heterogeneity in acquisition protocols and scoring systems limits comparability across centres. Quantitative MRI techniques and whole-body MRI offer promise but remain largely confined to research settings due to cost and feasibility considerations [45].

Muscle ultrasound is an attractive alternative because it is widely available, non-invasive, and well tolerated in children. Emerging evidence supports quantitative assessment of echogenicity, fascia thickness, and Doppler flow, which correlate with muscle function and disease activity [40–42, 46]. Nevertheless, operator dependence, limited standardisation, and variable sensitivity for deep muscle groups currently restrict widespread adoption [41].

### Histopathology scoring

A validated histopathology scoring system provides a clear and structured way to assess the changes seen in muscle biopsies from children with JDM, including inflammation, degeneration, vascular, and connective tissue involvement [47]. Although muscle biopsy is not routinely performed in clinical practice, this scoring system is especially helpful for research, prognosis, and standardized reporting in complex or difficult-to-treat cases. It has shown good reliability between different observers and across muscle samples, and modified versions focused on the most reliable features correlate well with clinical disease activity [47]. Studies have also found that more severe biopsy findings are linked to longer treatment needs over time [48].

### Patient-reported outcome measures

Generic PROMs like the CHAQ and PedsQL are commonly used in JDM and help clinicians and researchers understand how the disease affects children’s daily lives and well-being [9, 11, 13]. However, because these tools are not tailored to JDM, they can miss important details. For example, as motor function improves, many children approach the lowest possible CHAQ score (0), making it hard to distinguish those with ongoing milder symptoms.

PROMIS instruments are a newer option that efficiently assess fatigue, pain, physical function, and mental health. These tools have strong reliability and accuracy in juvenile myositis, and can better capture subtle differences in children with fewer symptoms, reducing the problem of scores bunching at the top or bottom [49–51]. They are available in computer adapted versions that shorten the time needed and make them more feasible for routine use. Still, there is a clear need for PROMs designed specifically for JDM, especially to better capture fatigue, how the disease affects children’s ability to take part in daily or social activities, and the burden of treatment.

### Subgroup and complication-specific assessments

Certain JDM complications require tailored assessment approaches. For example, lung involvement, such as interstitial lung disease (ILD), is usually evaluated with pulmonary function testing and imaging; in anti-MDA5-associated or otherwise ILD-prone JDM, interferon-related biomarkers may help identify patients who warrant earlier chest CT or closer pulmonary surveillance, and FDG-PET/CT has been explored as an adjunct marker of inflammatory activity in myositis-associated ILD, although its role in distinguishing activity from damage in paediatric JDM remains to be validated [52, 53]. Damage assessment is addressed by the Myositis Damage Index, which captures damage such as calcinosis, contractures, and metabolic complications [32, 54]. Long-term follow-up has demonstrated a high cumulative burden of damage across cutaneous, muscular, and skeletal domains, and early disease activity has been associated with later damage accrual [54]. Nailfold capillaroscopy provides an assessment of vasculopathy, and reduced capillary density and scleroderma-type patterns have been associated with disease activity [55]; however, standardised scoring systems specific to JDM remain limited.

A related challenge is defining and measuring inactive disease. The PRINTO criteria for clinically inactive disease (commonly used in research and increasingly referenced clinically) require at least three of four criteria: CK ≤ 150, CMAS ≥ 48, MMT8 ≥ 78, and PGA ≤ 0.2 [56–58]. Importantly, analyses suggest PGA ≤ 0.2 is particularly valuable to avoid classifying children with persistent skin disease as inactive, emphasising the value of PGA as a reliable measure for confirming inactive disease status [57].

## Recent advances: composite and novel clinimetric tools

In recent years, the field has witnessed significant progress in the development of composite clinimetric tools for JDM. The ACR/EULAR Total Improvement Score, for example, provides a continuous, weighted measure of clinical response, enhancing sensitivity and enabling a more nuanced assessment of treatment effects [7, 8]. This score ranges from 0 to 100, with established thresholds for minimal (≥ 30), moderate (≥ 45), and major (≥ 70) improvement. Its robust sensitivity and specificity have been demonstrated in JDM cohorts, supporting its use in clinical trials and comparative effectiveness research [8, 59, 60].

Another notable tool, the JDMAI, brings together core disease domains—such as physician and parent/child global assessments, muscle strength, and skin activity—into a single, integrated activity score [14]. Validation studies have shown this index to have strong construct validity, internal consistency, responsiveness to change, and the ability to distinguish across different levels of disease activity. Composite indices such as these are increasingly valued in both research and routine practice, as they streamline multidomain assessment while preserving the complexity of JDM. Alongside these, digital approaches including wearable activity monitors and remote PROMs collection are increasingly being explored to support longitudinal disease assessment. Emerging evidence from actigraphy and accelerometry studies in idiopathic inflammatory myopathies suggests that these tools may capture real-world physical activity and functional impairment and better detect fluctuations that may not be apparent during clinic visits [61, 62].

At the system level, the development of an international consensus optimal dataset for JDM, established through Delphi methodology with input from clinicians, patients, and families, represents a substantial advance. This comprehensive dataset of 123 items spans demographic, diagnostic, disease activity, and damage domains, and is expected to significantly enhance comparability across cohorts and support higher-quality multicentre research [10]. However, while the breadth of data collection is a major strength, initial experience from cohort studies suggests that the length and complexity of the dataset may contribute to clinician fatigue and an increased rate of missing data. This highlights the need for a pragmatic, streamlined version that could be more readily implemented in routine clinical practice without compromising data quality.

Emerging composite, patient-reported, and subgroup-specific clinimetric tools, along with their current limitations, are summarised in Table 2.

**Table 2:** Emerging and subgroup-specific clinimetric assessments in juvenile dermatomyositis

Tool	Domain	Purpose	Evidence / recent advances	Key gaps
ACR/EULAR TIS	Composite response	Continuous treatment response measure	Validated thresholds with high sensitivity/specificity	Data-intensive; mainly trial-focused
JDMAI	Composite disease activity	Single integrated disease activity score	Good validity and responsiveness	Cut-offs and routine use evolving
PROMIS	PROMs	Fatigue, pain, physical function, mental health	Validated in juvenile myositis; CAT reduces floor/ceiling effects	Not JDM-specific; participation/treatment burden gaps
MDI	Damage	Irreversible disease sequelae	Long-term cohorts show high damage burden	Captures cumulative damage rather than current activity; some items (e.g. calcinosis) may reflect ongoing inflammation.
MRI / WB-MRI	Imaging	Muscle, myofascial, and subcutaneous inflammation; subcutaneous abnormalities may predict calcinosis and chronic disease course	Sensitive detection; correlates with CMAS/MMT	Cost, access, protocol heterogeneity
Muscle ultrasound	Imaging adjunct	Non-invasive muscle assessment	Correlates with clinical activity	Operator dependence; lack of standardisation
ILD monitoring (PFTs, imaging)	Subgroup-specific	Lung disease assessment	Essential in affected patients	No validated JDM-specific index
Nailfold capillaroscopy	Vasculopathy	Microvascular assessment	Reduced capillary density linked to activity	No standardised JDM-specific scoring

Abbreviations: JDMAI, Juvenile Dermatomyositis Activity Index; JDMAR, Juvenile Dermatomyositis Multidimensional Assessment Report; PROMIS, Patient-Reported Outcomes Measurement Information System; CHAQ, Childhood Health Assessment Questionnaire; MDI, Myositis Damage Index; MRI, magnetic resonance imaging; WB-MRI, whole-body MRI; PFTs, pulmonary function tests; ILD, interstitial lung disease

## Treat-to-target (T2T) in JDM and its clinimetric foundations

T2T posits that aggressive pursuit of clinical targets leads to improved long-term health. In JDM, evidence-informed, consensus principles have recently been published [63]. The idea is that health-state targets are set, and treatment is adjusted if targets aren’t met within specific time frames [64]. To know whether a target is met, accurate measurements must be taken frequently.

## Unmet needs in JDM clinimetrics

Despite notable advancements in the development and validation of clinimetric tools for paediatric rheumatology, their consistent integration into everyday clinical practice remains elusive. Many of these instruments, while robust and comprehensive, are often time-consuming or require resources that are not universally available, thus limiting their practicality in busy or resource-limited settings. Assessment of skin disease continues to present significant challenges, as no single, universally accepted gold-standard tool has emerged to date [22, 23].

Moreover, important aspects of the patient experience—such as fatigue, the ability to engage in daily and social activities, and the overall burden of treatment—are not adequately addressed by current measures. The absence of disease-specific PROMs persists as a major gap, even as PROMIS instruments have improved the scope and sensitivity of patient assessment [13, 49–51]. Additionally, there remains a lack of standardised approaches for monitoring ILD and for the early identification of organ involvement, both of which are critical to comprehensive care.

Global disparities in access to advanced imaging modalities and biomarker testing represent a persistent barrier, further complicating the widespread adoption of these tools and highlighting the need for scalable, context-appropriate solutions. Addressing these challenges will require ongoing, collaborative international efforts to develop pragmatic and resource-sensitive strategies that can be implemented across diverse healthcare settings. Central to this process is the active involvement of clinicians, patients, and families in the iterative refinement of assessment tools, ensuring they are both scientifically rigorous and truly reflective of real-world needs. In this context, digital health innovations hold significant promise for bridging gaps in access and streamlining data collection, particularly in underserved regions.

## Future directions

Looking ahead, the future development of JDM clinimetrics should prioritise the creation of disease-specific PROMs, with particular attention to domains such as fatigue and the ability to participate in everyday life and social activities. In parallel, efforts should focus on the integration of validated biomarkers—such as interferon-related markers and other emerging molecular signatures—into outcome frameworks as the evidence base continues to grow [33, 34]. Advances in imaging, artificial intelligence, and digital phenotyping are expected to further enhance objectivity and enable more robust longitudinal monitoring, including the harmonisation of MRI scoring and the development of standardised ultrasound protocols. Ultimately, the integration of clinical, biological, and patient-reported data into unified, multidimensional indices will be central to advancing precision medicine for children affected by JDM.

## Conclusions

There has been tremendous international effort, and success, in developing instruments for JDM health measurement. Best-practice treatment strategies, e.g., T2T, rely on frequent and accurate measurements. Our goal going forward is to continue this work to provide even better, more feasible, measures with strong input from patients; a simple, single, unified tool that captures all the important aspects of health is the goal.

## Data Availability

No datasets were generated or analysed during the current study.
